# Involving general practice trainees in clinical practice guideline adaptation

**DOI:** 10.1186/s12909-018-1252-9

**Published:** 2018-06-22

**Authors:** Nicolas Delvaux, Martine Goossens, Paul Van Royen, Stijn Van de Velde, Robert Vanderstichele, Hanne Cloetens, Jan Vanschoenbeek, Bert Aertgeerts

**Affiliations:** 1 Department of Public Health and Primary Care, KU Leuven, Kapucijnenvoer 33, Blok J, PB 7001, B-3000 Leuven, Belgium; 2 Department of Primary and Interdisciplinary Care, University of Antwerp, Antwerp, Belgium; 3 Division for Health Services, Norwegian Institute of Public Health, Oslo, Norway; 4 Department of Pharmacology, Ghent University, Ghent, Belgium; 5 Flemish College of General Practitioners, Domus Medica, Antwerp, Belgium; 6 EBMPracticeNet, Leuven, Belgium

**Keywords:** Clinical practice guidelines, Guideline adaptation, General practice, Education

## Abstract

**Background:**

It is unclear whether it is feasible to involve residents in guideline development or adaptation. We designed a multifaceted training program that combines training sessions, a handbook and a documentation tool to assist general practice (GP)-trainees in the adaptation of clinical practice guidelines (CPGs). The aim of this study is to adapt a database of CPGs by involving GP-trainees and to build evidence-based practice (EBP) learning capacity.

**Methods:**

We assessed each adaptation process and surveyed all GP-trainees who enrolled in our training program on their views on the program. They were asked to formulate an overall rating for the training and were asked to rate individual aspects of the training program (the training sessions, the handbook and the documentation tool).

**Results:**

To date, 122 GP-trainees followed the training and have adapted 60 different CPGs. Overall quality of their work was good. Based on an assessment of the content of the documentation tool, 24 (40%) adapted CPGs rated as good quality and 30 (50%) rated as moderate quality. Only 3 adapted CPGs (5%) were evaluated as being of poor quality. 51 (42%) GP-trainees completed the survey on user satisfaction. 98% (50) of the GP-trainees found the training to be of good overall quality. 86% of the GP-trainees were satisfied with the handbook but satisfaction was lowest for the documentation tool (47% satisfied).

**Conclusion:**

It is possible to engage GP-trainees in CPG adaptation using a formal process when provided with training, feedback and documentation tools.

**Electronic supplementary material:**

The online version of this article (10.1186/s12909-018-1252-9) contains supplementary material, which is available to authorized users.

## Background

Medical schools are embracing evidence-based medicine (EBM) as a core competence for future doctors [1]. Key for this competence include having the knowledge, skills and aptitudes to formulate a focused health question, perform a literature search, critically appraise evidence, apply it to the clinical context, and evaluate the outcome of the clinical decision for the individual patient [2]. Clinical practice guidelines (CPGs), defined as “statements that include recommendations intended to optimize patient care that are informed by a systematic review of the evidence and an assessment of the benefits and harms of alternative care options” [3], are not only an important source of EBM information for practicing clinicians, but also increasingly influence medical education. However, to understand and critically review recommendations from CPGs, a profound acquirement of these EBM competences is necessary. This includes a thorough understanding of how to search, how to evaluate the quality, when to apply, and also important, when not to apply a recommendation [4]. In Belgium, medical schools provide training in the critical appraisal of CPGs and this is evaluated through multiple choice tests. However it is unclear whether residents master these skills on a performance or action level as defined by Miller, upon graduation [5]. An Australian program has shown that training and involving medical students in the development of Cochrane systematic reviews resulted in publishable reviews, indicating a high degree of competence [6]. To our knowledge, no similar programs exist that describe the involvement of medical students or residents in CPG development or adaptation.

In 2011, Belgium acquired a national license for EBM Guidelines, a database of point-of-care (POC) guidelines, covering around 1000 topics relevant to primary care medicine [7]. Some guidelines in this database required adaptation to be fully applicable in the Belgian context. Guideline adaptation, defined as the “systematic approach to the endorsement and/or modification of guidelines produced in one cultural and organizational setting for application in a different context”, has shown to be a less resource-demanding method for developing high quality guidelines [8, 9]. This prompted us to explore the feasibility of recruiting general practice (GP)-trainees, on a yearly basis to assist in this task as part of their vocational training. We designed a multifaceted program that combines training sessions, a handbook and a documentation tool to train them in CPG adaptation. The aim of this study was to adapt the EBM Guidelines by involving GP-trainees and to build evidence-based practice (EBP) learning capacity through a multifaceted training program. In this paper we describe the adaptation process and training program used in this study and evaluate GP-trainee satisfaction with the training program and EBP learning.

## Methods

### Setting

In Flanders, vocational training in primary care medicine (or family medicine) consists of a three-year postgraduate master degree. The Dutch speaking universities of Flanders offer this degree. These four universities have decided to organize this curriculum and the trainings in collaboration through a common institute. Each year around 300 new GP-trainees are enrolled in this postgraduate degree, which includes classroom teaching, internships and a master paper. Our program was designed in support of the master paper which is completed in the last two years of the vocational training, and was not part of the formal curriculum.

### Intervention

**Fig. 1:**
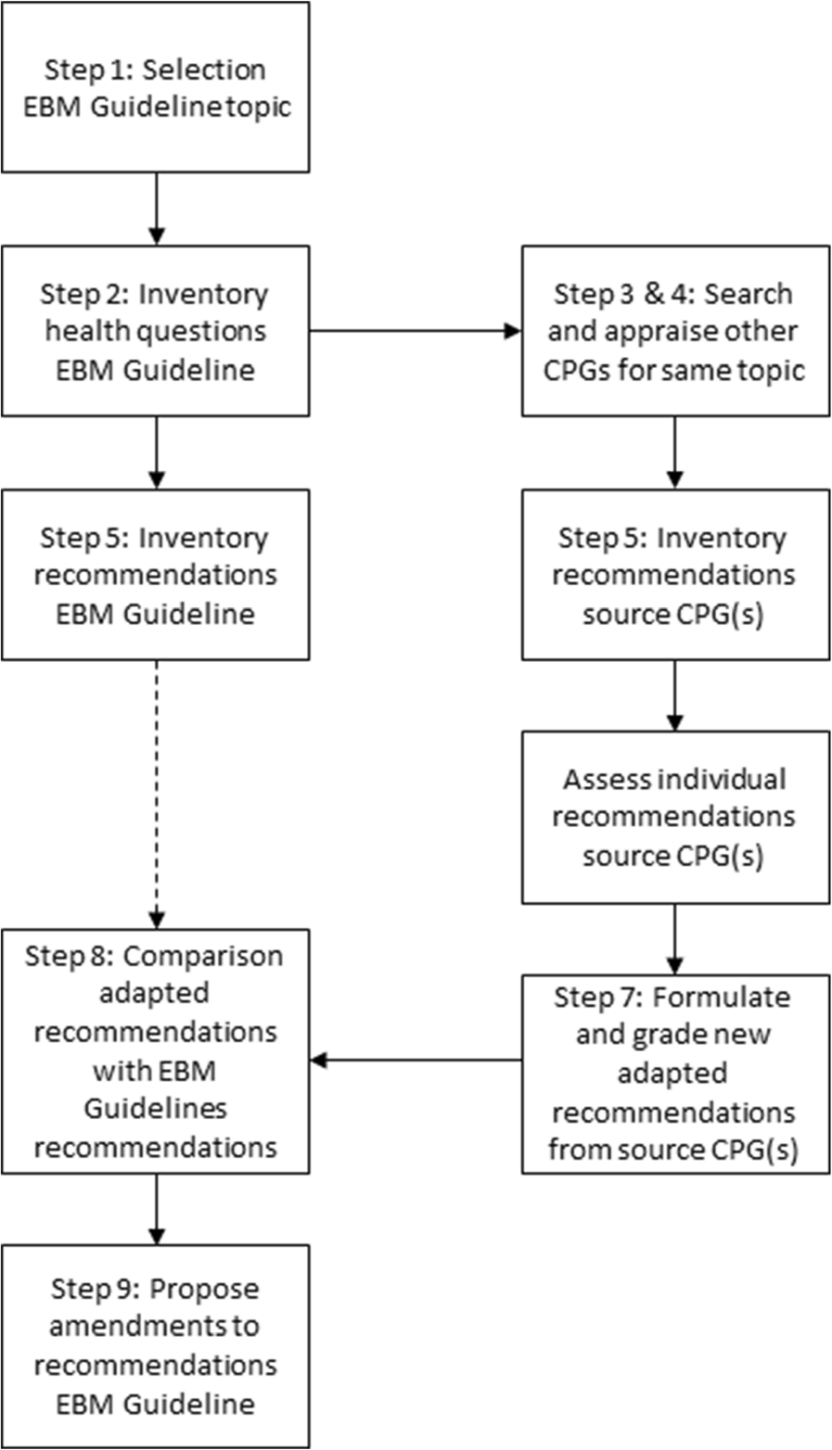
Overview of the adaptation process. Details of each step are described in Table 1. EBM: evidence-based medicine; CPG: clinical practice guideline

In order to correctly determine whether recommendations are applicable in a context other than the one for which they were developed, a thorough assessment of at least the evidence base, the trade-offs of benefits and harms, and local preferences is required. Because of their summarized nature, the EBM Guidelines did not provide the necessary information to conduct this assessment and therefore could not be used as source CPGs for adaptation. We designed a process, based on the ADAPTE framework [9, 10], through which other high quality source CPGs on the same topic and their recommendations were adapted to the Belgian context. These adapted recommendations were then compared with the recommendations in the original EBM Guideline. Figure 1 illustrates the steps of this process and the details of each step are described in Table 1. Many of these steps are complicated and most GP-trainees were unable to do these without prior training. Hence, we designed a training program which included:

- A handbook written to guide the GP-trainees through the various steps of the adaptation process,
- A documentation tool (or matrix) designed in an Excel worksheet that allowed the GP-trainees to document each step in a uniform and transparent way (see Additional file 1: Appendix 1 for an example of the matrix),
- Five interactive group sessions lasting two hours, during which the different steps of CPG adaptation were discussed and the results of previous steps were reflected upon.

**Table 1:** Overview of the various steps included in the adaptation process

	Step of the adaptation process	Full description
1	Selection of a guideline topic from the database of EBM Guidelines	From the database of more than 1000 EBM Guidelines a shortened list was generated from which GP-trainees could select a topic. This list was designed to prevent GP-trainees from choosing unsuited topics based on relevance for primary care and availability of trustworthy evidence
2	Identification and framing of the health questions addressed in the EBM Guideline	EBM Guidelines do not provide a list of addressed health questions. We therefore instructed GP-trainees to identify them based on the recommendations formulated by EBM Guidelines. We also instructed them to frame the health question using the PICO or PIPOH format
3	Performing a literature search, including source CPGs	GP-trainees were instructed to perform a literature search for source CPGs on the guideline topic
4	Assessment of the quality of source CPGs using the AGREE II instrument and selection of one or more source CPGs based on this assessment	Each of the source CPGs was assessed for quality using the AGREE II instrument [12]. Only those source CPGs rated as high quality using this instrument were considered as source CPG for further adaptation
5	Identification of recommendations in the source CPGs	For each source CPG, all recommendations related to the formulated health questions were inventoried in the matrix
6	Assessment of currency, consistency and applicability of recommendations in the source CPGs	All of the inventoried recommendations were assessed for currency, consistency and applicability. A literature search for more recent primary studies not included in the evidence review of the source CPG was performed to assess whether the recommendations were sufficiently up to date. Additionally, for each recommendation an evaluation of the consistency between the recommendation and the cited evidence was made. Finally, based on own experience and knowledge of the healthcare system, an assessment of the local applicability of the recommendation was made
7	Formulating (adapted) recommendations based on the results of the previous step and grading of the recommendations	Based on the previous assessments, recommendations from the source CPGs were either adopted without changes, adapted with changes or omitted. GP-trainees graded the evidence and the recommendations using GRADE.
8	Comparison of the newly formulated (adapted) recommendation with the recommendations in the EBM Guideline	These recommendations (adapted from source CPGs) were then compared with the recommendations formulated by EBM Guidelines. Any inconsistencies were documented including the underlying rationale
9	Proposal of amendments to recommendations in the EBM Guideline based on the comparison	When GP-trainees encountered inconsistencies between their adapted recommendations and the recommendations in the EBM Guideline, they were instructed to propose an amendment to the EBM Guideline. Inconsistencies due to new evidence or flaws in the evidence review were relayed back to the editorial board of EBM Guidelines. Inconsistencies due to applicability, differences in practice, preferences or regulations resulted in an amended recommendation on the EBPracticeNet website.
10	Implementation of the (adapted) recommendation and evaluate the implementation as part of a quality improvement strategy	GP-trainees were instructed to evaluate the applicability and implementability of the (adapted) recommendations through a quality improvement strategy. The purpose for this task was to provide more information on the applicability of the recommendations and to document barriers or facilitators to adherence to the recommendations. Sometimes this step resulted in an amendment to the recommendations to improve applicability

*AGREE* Appraisal of Guidelines for Research and Evaluation, *CPG* clinical practice guideline, *PICO* Population, Intervention, Comparator, Outcome, *PIPOH* Population, Intervention, Profession, Outcome, Healthcare setting, *GRADE* Grading of Recommendations Assessment, Development and Evaluation

**Fig. 2:**
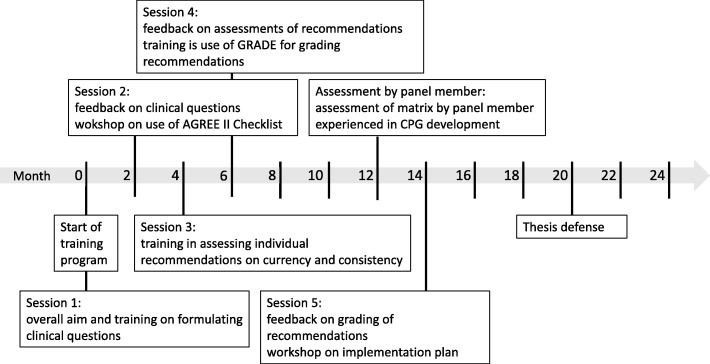
Timeline of the different steps of the two year training program. Month 0 corresponds to the beginning of second year of the three year vocational training. AGREE: Appraisal of Guidelines for Research and Evaluation; CPG: Clinical practice guideline; GRADE: Grading of Recommendations Assessment, Development and Evaluation

Figure 2 illustrates the overall training program and Table 2 summarizes the content of each session+. The first session of the training was organized at the start of the two year program. We informed GP-trainees on the aim and overall process of the training. We included a one hour training on how to formulate health questions and how to translate these into a PICO (Population, Intervention, Comparison, Outcome) or PIPOH (Population, Intervention, Professionals targeted, Outcome, Healthcare setting) depending on the scope of the health question, and on how to search for literature [11]. The training focused on searching for CPGs and synthesized evidence such as systematic reviews pertaining to their health questions, rather than primary studies. GP-trainees were instructed to form pairs and to select an EBM Guideline topic. The GP-trainees were given two months to formulate at least two health questions each and to conduct a first literature search. Each pair was advised to invite a promotor to assist them in the adaptation process and the writing of the final master paper.

**Table 2:** Summary of the five training sessions

Session 1	
2 h Start	Explanation of overall aim and scope of training program
	Training on formulating health questions, use of PICO and PIPOH
Session 2	
2 h Month 2	Feedback on health questions
	Workshop on the use of AGREE II for assessing CPG quality
Session 3	
2 h Month 4–5	Training on the assessment of individual recommendations with focus on currency, consistency and applicability
Session 4	
2 h Month 6–7	Feedback on assessments of individual recommendations
	Training on the use of GRADE for grading of evidence and recommendations
Session 5	
2 h Month 11–12	Feedback on GRADE
	Workshop on quality improvement strategies or implementation plans

*PICO* Population, Intervention, Comparator, Outcome, *PIPOH* Population, Intervention, Professional, Outcome, Healthcare setting, *AGREE* Appraisal of Guidelines for Research and Evaluation, *CPG* Clinical practice guideline, *GRADE* Grading of Recommendations Assessment, Development and Evaluation

After these two months, during a second session, a further reflection was made of the health questions and the PICO or PIPOH versions of these questions in a one-hour interactive workshop. In the second hour of the training session, we instructed the GP-trainees on the use of the Appraisal of Guidelines for Research and Evaluation (AGREE) II instrument for the appraisal of CPG quality [12]. During a subsequent two to three month period, GP-trainees were instructed to use this instrument to assess the quality of potential source CPGs and to identify all the key recommendations relevant to their health questions.

The third session focused entirely on illustrating how to asses individual recommendations on currency and consistency. This session instructed GP-trainees how to assess the currency of formulated recommendations and the consistency between a recommendation and the supporting evidence with the evaluation of harms and benefits. Additionally, GP-trainees were familiarized with methods to evaluate whether the results of primary studies were consistent with the way the authors of source CPGs summarized the evidence.

Two months later, in a fourth session, we provided feedback on the currency and consistency assessments in a workshop. We used examples from the GP-trainees’ matrices to provide advice on common mistakes or problems. During the following 90 min GP-trainees were introduced to methods for assessing quality of evidence and strength of recommendation using the Grading of Recommendations Assessment, Development and Evaluation (GRADE) method [13].

At the end of the second year, when the GP-trainees had completed the adaptation process and had succeeded in formulating evidence-based recommendations for the defined health questions, these were compared with the recommendations in the EBM Guideline. In case of important inconsistencies due to flaws in the analysis or interpretation of the supporting evidence, a summary of the findings was reported to the editorial board of EBM Guidelines. In case of inconsistencies pertaining to issues of applicability, i.e. differences in local availability of drugs, different statistics on pathogen resistance to antibiotics, differences in local legislation, etc., a change in the original recommendation was proposed to amend the recommendation in the original EBM Guideline in order to better apply to the local setting.

In a final session, at the start of the third year, we provided feedback on the grading of the quality of evidence and the assessment of the strength of recommendation using the information provided by the GP-trainees. We asked each GP-trainee to present their plans, including quality improvement strategies or implementation plans, to assess the applicability of the recommendations they formulated. These plans often involved practicing GPs, clinicians or other healthcare practitioners. Table 1 summarizes the content and focus of each of these sessions.

To support each GP-trainee in writing their master paper, promotors were invited to attend a training on guideline adaptation so that those with little or no experience in methods for guideline adaptation would be better informed on the various steps in the process.

### Outcomes

We used Kirkpatrick’s four-level evaluation model to inform the evaluation of our program [14, 15]. We aimed to assess the satisfaction and reactions of the participating GP-trainees to our program, the first level of this evaluation model. For this, GP-trainees involved in our training program were surveyed for their satisfaction. They were asked to formulate an overall rating for the training on a three point scale (good, moderate or poor) and were asked to rate individual aspects of the training program (the training sessions, the handbook and the matrix). We also received remarks and comments which were used to improve and tailor the training program over the years.

In addition, we wanted to measure effect of our program on the measure of learning (the second level of Kirkpatrick’s evaluation model), which we evaluated by the quality of the adapted CPGs and the proposed revisions to specific recommendations. For this we asked a panel of eight experts in CPG development to appraise the quality of the GP-trainees’ adapted CPG using a checklist we developed based on the AGREE II instrument (see Additional file 2: Appendix 2), but focused on the methodology of the process and tailored to assess each step of the guideline adaptation process. We chose to use this checklist because it was already in use for the evaluation of adaptation processes in a previous study [9], and the evaluators were already experienced in its use. The checklist was not validated against a gold standard, but was piloted by experienced CPG developers and found to be reliable in guiding the evaluation process. Each GP-trainee submitted their matrix to one member of this panel for evaluation and received feedback before commencing with their quality improvement strategy or implementation plans. We chose to evaluate the matrix rather than the actual adapted CPG because the matrix more clearly demonstrated whether the GP-trainees correctly performed all the steps of the adaptation process. Based on the results of the checklist, a subjective assessment of the matrices was made, with as result either good, moderate or poor (comparable with the results of the AGREE II instrument). A matrix was considered good if for each recommendation all steps of the adaptation process were correctly documented, moderate if some of the steps were insufficiently documented of not correctly performed and poor if important elements of the process were lacking or not correctly performed.

The implementation plans presented by each GP-trainee during the final session provided an opportunity for measuring changes in behavior (third level of Kirkpatrick’s evaluation model) or even resulting clinical practice (fourth level of the evaluation model), but due to the diversity in these implementations plans and results, it proved to be impossible to use these as outcome measures.

## Results

The training program was first introduced in 2012, and each year on average 24 GP-trainees enrolled in our training program. Recently, 20 new GP-trainees have elected to start our training program in 2017. To date, 122 GP –trainees followed the training and evaluated 60 different EBM Guideline topics by comparing them with an adapted version of high quality source CPGs.

Over the last five years, 51 of the 122 GP-trainees completed the survey on user satisfaction (see Table 3 for the results of the survey on user satisfaction). Most GP-trainees (50) found the training to be of good overall quality. 86% of the GP-trainees were satisfied with the handbook. Satisfaction with the use of the matrix was limited to 47%. With regard to the training session, 8 in 10 GP-trainees were satisfied with the frequency of sessions, with the time spent, and with the content on the sessions.

**Table 3:** Results GP-trainee satisfaction survey

Rating		Good	Moderate	Poor
Overall satisfaction, *N* (%)		50 (98)	–	1 (2)
Satisfaction handbook, *N* (%)		44 (86)	5 (10)	2 (4)
Satisfaction matrix, *N* (%)		24 (47)	25 (49)	2 (4)
Satisfaction training sessions	Frequency of the sessions, *N* (%)	45 (88)	4 (8)	2 (4)
	Time well spent, *N* (%)	45 (88)	3 (6)	3 (6)
	Content of the sessions, *N* (%)	43 (84)	–	8 (16)

Throughout the Six years that we have organised this training, we received useful comments and suggestions. Several GP-trainees complained about the inconsistency between the advice given during the training sessions versus the advice given by their promotor. When evaluating these complaints, often the conflicts were rooted in the fact that the promotor had expertise in the CPG topic, but not in CPG development methodology.

Most GP-trainees were satisfied with the content of the sessions, but relevant comments concerned the training on formulating health questions. In proportion to the whole process, most time was spent on this, but still this step was most often perceived as difficult often additional coaching outside of the training sessions was needed. Especially health questions on diagnostics, indications for referral and situations warranting follow-up tended to be difficult for GP-trainees. Additional file 3: Appendix 3 shows some examples of health questions and highlights some of the difficulties GP-trainees experienced.

GP-trainees were least satisfied with the Excel matrix (47% found the matrix to be good). It was designed to aid GP-trainees in documenting all relevant steps in the adaptation process and to aid in the review of their work but was not ideally suited for this purpose.

Of the 122 completed documentation tools (matrices), 119 were evaluated using our checklist. Of these matrices, 24 (40%) were considered of good quality by the reviewers. Thirty matrices (50%) were considered of moderate quality. Only 3 matrices (5%) were evaluated as being of poor quality. All three of these matrices date from the first two years after the start of the training program. The reasons for their poor evaluation were insufficiency in the rigor in assessing the consistency of the individual recommendations and insufficiency in the documentation of the process in the matrix. For 3 matrices (5%), no review was made or the results were lost. All GP-trainees successfully completed their master thesis, which also included an evaluation of their quality improvement or implementation studies.

## Discussion

Educational experiences and building capability in EBM has been shown to be influential towards residents’ intention to use EBM after graduation [16]. However, few teaching strategies have succeeded in including all steps considered part of the EBM process [17] and it remains unclear which strategies are most effective in attaining EBM skills [18–20]. Our study demonstrated that GP-trainees, with limited prior experience in EBM, can effectively perform all of the steps of the EBM process through CPG adaptation, when provided sufficient training, feedback and documentation tools. Even though it has been suggested that classical classroom EBM training appears more effective than problem-based learning [20], other studies suggest that it should be embedded in a hands-on training [21] or as part of a multifaceted approach to training [18]. Several examples of hands-on training have been used in curricula, such as critically appraised topics (CATs) [22], Morning Reports, Journal Clubs [2] and involvement in Cochrane Reviews [6]. Our study showed that EBM training as part of a more comprehensive process such as CPG adaptation can be added to this array. We chose to involve GP-trainees in our program, because clinical experience is important to correctly understand the context in which recommendations from CPGs are to be applied. There are differing views on when teaching EBM skills should be introduced to curricula, but a growing consensus appears to be emerging that several basic research skills must be mastered first [18]. Hence, even though our program proved feasible for GP-trainees, it may not be in undergraduate curricula.

In the evaluation of our program, overall satisfaction was high and GP-trainees found it, including training sessions, handbook and matrix to be useful and acceptable. So far all, but one, GP-trainees successfully completed the training indicating high participant engagement. A review of the adapted guidelines found that the large majority of adapted CPGs were of moderate to high quality indicating that GP-trainees can successfully complete the process. The original aim of our study was to involve GP-trainees in CPG adaptation to increase the capacity to amend the EBM Guidelines available on the EBPracticeNet platform to better suit the Belgian context. For this task we amended EBM Guidelines based on the comparisons with adapted CPGs of high and moderate quality. Although the EBPracticeNet platform strives for high quality, a recent systematic review of 415 CPGs found that 37% scored as ‘recommended’ and 45% scored as ‘recommended with modifications’ indicating that even experienced CPG developers have difficulty in developing high quality CPGs [23].

Several lessons were drawn from this study which allowed us to develop new strategies to improve our training program. We found that it was important to provide training for promotors alongside training for GP-trainees. We encouraged all promotors to attend a training on methodology but few promotors were able to attend these sessions. Busy schedules and differences in attitude regarding the need for rigorous methods for guideline development were important barriers to this strategy. In later years, per faculty, an experienced CPG developer was designated as contact person for methodological issues. This person was often solicited as co-promotor by the GP-trainees for more individual guidance on methodological issues. This improved, but did not end, remarks regarding conflicts in advice between the promotor and the trainings. In order to be effective CPGs should, where possible, provide evidence-based answers to real-life health questions posed by physicians [24]. In CPG development, an important challenge is being able to translate these real-life health questions into clearly defined research questions. Many processes in CPG development rely on a PICO-centred model [25]. It is therefore important that GP-trainees correctly define their health questions before continuing in the process of CPG adaptation. It is difficult to find the balance between too narrow and too broad health questions, and this is often an iterative process, especially for less experienced participants [26]. Involving someone with clinical expertise and a good understanding of the available research can improve the quality of the health questions. More specifically, questions on diagnostics, indications for referral and situations warranting follow-up tended to be difficult for GP-trainees. Individual feedback was very often instrumental to improve the quality of the health questions.

The use of technological tools and online materials in EBM teaching is still in its infancy, but previous studies have shown some promising results [18]. Our study demonstrated that a tool to collect all the work and to document each step of the process is essential to guide the GP-trainees and to facilitate the review of the work. It is important that this tool allows multiple users to work on the same project and should be designed to ensure a certain degree of uniformity, not only in methods or processes used, but also in output and reporting. Our matrix was not able to fully address these needs. We experimented with the guideline authoring tool MAGICapp [27] as an alternative with two GP-trainees, but found that GP-trainees required a more detailed understanding of grading of evidence. We did notice that the use of this tool appeared to result in a clearer understanding of assessing quality of evidence and better transparency in GP-trainees’ reporting.

Our study presents several limitations. First, our study was designed to inform the quality improvement of our program rather than evaluate its effectiveness. For the evaluation of the adaptation quality we used a specifically designed checklist with a subjective outcome based on the results of the checklist. Our checklist was not validated or compared with existing instruments, such as the AGREE II instrument. However, the focus of the assessment was not so much the quality of the adapted CPG but to evaluate whether the GP-trainees successfully mastered the EBM skills required for CPG adaptation. This limited our evaluation to the results of the first year of the two year program. We considered using the assessment of the master thesis as additional evidence that the learning objectives were achieved, however this assessment was not made by methodological experts in CPG development and hence less reliable. We chose to evaluate GP-trainee’s progress by one experienced CPG developer rather than by two or more reviewers, as would be customary in a research design. The assessors collaborated in this study voluntarily and it was not feasible to request a larger investment from them. The subjective character of our checklist may compromise reproducibility to other settings, but the absence of a uniform, reliable instrument to assess EBM competency remains a challenge in measuring effectiveness of EBM teaching strategies [19, 28]. A systematic review of EBM tests used in GP-trainees showed that most evaluations were restricted to measuring basic EBM skills such as identifying PICO elements, but recognized the limitations of these evaluations when including CPGs in EBM courses [29]. We believe the subjectivity of our checklist should not influence the results significantly because inter-rater agreement in the use of the AGREE instrument (which our checklist is based on) by experienced users has been shown to be high [23, 30]. Moreover, as stated before, the assessors were methodological experts in CPG development.

Second, we did not evaluate the feasibility of our program for the supervisors and assessors. Our program is largely reliant on the dedication of the supervisors providing the training sessions and the individual feedback. This proved to be a time-consuming task and caused us to limit the number of GP-trainees to 20 per year. Increasing the number of participating GP-trainees resulted in less detailed feedback during the training sessions and sometimes left little time over to train them in the next steps of the process. Organizing the training for larger groups will require a substantial amount of supervision, but our study could not provide correct data on the exact time investment.

A third limitation to our study was the low response to our survey by GP-trainees. Surveys were administered and collected during the last training session, except for once in 2015 when it was administered by mail. That year, we collected only one completed survey, which largely explains the low response rate. The response rates in the other years was much higher and motivated us to continue collecting the surveys during the last training session.

## Conclusions

This study demonstrated that it is feasible to engage GP-trainees in CPG adaptation using a formal process when provided with training, feedback and documentation tools. Our study showed that it is crucial to involve experienced CPG developers as supervisors and members of the evaluation panel, however the time investment required for this task may be an important barrier. In addition, a clear focus is needed on identifying and formulating health questions to be addressed by the CPG. Further research is needed to determine whether student involvement might also be instrumental to resource intensive activities such as CPG development and updating.

## Additional files

Additional file 1: Appendix 1 Example of matrix. An example of the documentation tool provided for GP-trainees to document all their steps of the CPG adaptation process. (XLSX 155 kb)

Additional file 2: Appendix 2 Evaluation tool. The evaluation tool, based on the AGREE instrument, and tailored to assist in the evaluation of the CPG adaptation process. (XLSX 54 kb)

Additional file 3: Appendix 3 Examples of health questions. Several examples of difficulties GP-trainees experienced in formulating health questions. (DOCX 18 kb)

## Abbreviations

AGREE: Appraisal of Guidelines for Research and Evaluation
CAT: Critically appraised topic
CPG: Clinical practice guideline
EBM: Evidence-based medicine
EBP: Evidence-based practice
GP: general practitioner
GRADE: Grading of Recommendations Assessment, Development and Evaluation
PICO: Patient, Intervention, Comparison, Outcome
PIPOH: Patient, Intervention, Professional, Outcome, Healthcare setting
POC: Point-of-care
