# Supplementary material for: Involving general practice trainees in clinical practice guideline adaptation
Source: BMC Med Educ. 2018 Jun 22;18:148. doi: 10.1186/s12909-018-1252-9 (PMC6013901; doi:10.1186/s12909-018-1252-9)
Supplement: Supplementary file 3 — Appendix 3 Examples of health questions. Several examples of difficulties GP-trainees experienced in formulating health questions. (DOCX 18 kb) [file 12909_2018_1252_MOESM3_ESM.docx]

# Examples of clinical questions and PIPOH elements for guideline adaptation

In these examples, we instructed GP-trainees to formulate a narrative health question (what is the question a physician would have at the point of care when confronted with a patient?) and to identify the PIPOH elements required to perform a structured literature search. PIPOH is the acronym for Population, Intervention, Professionals, Outcomes and Healthcare Setting. We used the PIPOH approach as opposed to the more common PICO (Population, Intervention, Comparison, Outcomes) approach because in clinical practice guideline (CPG) adaptation, comparison of health questions between source guidelines can be complicated by narrow PICOs and it is important to include the context in which the studies were performed.

# Health questions on risk factors

## Which risk factors (I) influence the development of disease (O) in population (P)?

### Correct example

**In which people with bite wounds are antibiotics indicated in primary care?**

| PIPOH elements | Inclusion criteria | Exclusion criteria |
| --- | --- | --- |
| Patient/Population | Patients with bite wound | Patients without bite wound |
| Intervention(s) | Risk factors for complicated bite wounds, infections |  |
| Professionals | Not specified |  |
| Outcomes | Prescription of antibiotics, hospitalization, specialized care |  |
| Healthcare Setting | Primary care |  |

In this example, the PIPOH elements will correctly identify studies that investigate risk factors for complications due to bite wounds. The presence of these risk factors could warrant the use of antibiotics.

### Incorrect example

**What are the indications for post-exposure prophylactic antibiotic treatment in people who were in close contact with a patient with whooping cough?**

| PIPOH elements | Inclusion criteria | Exclusion criteria |
| --- | --- | --- |
| Patient/Population | Person with close contact with patient with whooping cough | No laboratory confirmed pertussis |
| Intervention(s) | Antibiotics | Non-pharmacological treatment |
| Professionals | Physicians |  |
| Outcomes | Incidence of whooping cough |  |
| Healthcare Setting | Primary care, secondary care |  |

In this example, the PIPOH elements will identify studies that investigate the effectiveness of antibiotics for post-exposure prophylaxis of whooping cough. However, the health question should also guide a search for studies that identify populations where the risks of developing complications due to whooping cough are greater.

# Health questions on diagnosis

## In patients with symptom (P), how accurate is test (I) in diagnosing disease (O) compared to a gold standard?

### Correct example

**Which (additional) tests which can be performed in primary care are indicated in patients with IBS symptoms?**

| PIPOH elements | Inclusion criteria | Exclusion criteria |
| --- | --- | --- |
| Patient/Population | Patients with symptoms of IBS (diagnostic criteria) including one or more defining criteria |  |
| Intervention(s) | Any laboratory tests, fecal tests | Ultrasound, colonoscopy, gastroscopy |
| Professionals | Primary care physicians |  |
| Outcomes | Diagnosis other than IBS (coeliac disease, IBD, etc.) |  |
| Healthcare Setting | Primary care |  |

This health questions correctly identifies studies that use non-invasive diagnostic tests which can be used in primary care to identify patients with symptoms of IBS, but who have another underlying disease.

### Incorrect example

**What are the diagnostic criteria for Meniere disease?**

| PIPOH elements | Inclusion criteria | Exclusion criteria |
| --- | --- | --- |
| Patient/Population | Patients with Meniere disease |  |
| Intervention(s) | Diagnostic tool |  |
| Professionals | Physicians (primary or secondary care) |  |
| Outcomes | Diagnosis of Meniere disease |  |
| Healthcare Setting | Primary care |  |

The PIPOH elements of this health question are not correct. The population in which this diagnostic tool is assessed should be patients with suspicion of Meniere disease. Moreover, the health question is too ambiguous and should specify whether studies on clinical signs are included or whether only technical investigations are included.

# Health questions on interventions

## In patients with condition (P), what is the effect of intervention (I) on outcome (O) compared to (C)?

### Correct example

**Should vitamin D supplements and/or calcium supplements be offered to patients with elevated risk for osteoporosis?**

| PIPOH elements | Inclusion criteria | Exclusion criteria |
| --- | --- | --- |
| Patient/Population | Patients with elevated risk for osteoporosis (defined by formal risk assessment) | Studies including all women above certain age |
| Intervention(s) | Calcium supplements and/or vitamin D supplements |  |
| Professionals | Physicians |  |
| Outcomes | Fractures, hospitalization, mortality |  |
| Healthcare Setting | Primary care, secondary care |  |

This health questions and the corresponding PIPOH elements correctly identify studies that investigated the effect of calcium and/or vitamin D supplements in patients with an elevated risk for osteoporosis.

### Incorrect example

**How long should a drug treatment for restless legs syndrome be continued?**

| PIPOH elements | Inclusion criteria | Exclusion criteria |
| --- | --- | --- |
| Patient/Population | Patients with RLS |  |
| Intervention(s) | Any drug treatment | Non-pharmacological treatment |
| Professionals | Physicians |  |
| Outcomes | Duration of treatment |  |
| Healthcare Setting | Primary care, secondary care |  |

This health question is problematic because it is in fact part of the health question on the effectiveness of drug treatments on RLS. Studies on drug treatments for RLS will be able to provide evidence on treatment duration, but will never have ‘duration of treatment’ as an outcome. This health question should be formulated to search for studies on the effectiveness of certain drug treatments on RLS rather than on duration of treatments.

# Health questions on referral or follow-up

These health questions were very difficult for GP-trainees because the intervention will never be the follow-up or the referral. These actions will facilitate another intervention and clinical reasoning on why follow-up is organized or why a patient would be referred is required.

### Correct example

**When is referral for additional investigations in secondary care (to exclude other diseases) indicated in patients with IBS symptoms?**

| PIPOH elements | Inclusion criteria | Exclusion criteria |
| --- | --- | --- |
| Patient/Population | Patients with symptoms of IBS (diagnostic criteria) including one or more defining criteria |  |
| Intervention(s) | Intolerance tests, ultrasound, endoscopy, DNA tests, etc. | Diagnostic tests that can be performed in primary care |
| Professionals | Physicians, gastro-enterologists |  |
| Outcomes | Exclusion of other diseases such as coeliac disease, lactose-intolerance, IBD, polyps, diverticulitis, etc. |  |
| Healthcare Setting | Secondary care |  |

### Incorrect example

**What follow-up investigations are indicated in patients with Meniere disease?**

| PIPOH elements | Inclusion criteria | Exclusion criteria |
| --- | --- | --- |
| Patient/Population | Patients with Meniere disease |  |
| Intervention(s) | Follow-up investigations |  |
| Professionals | Physicians |  |
| Outcomes | Improvement of symptoms, ADL |  |
| Healthcare Setting | Primary care |  |

The PIPOH elements of this health question demonstrate a lack of insight in study designs. Follow-up investigations are never interventions, but a means to evaluate treatment effect, identify adverse effects at an early stage, etc. To correctly identify these PIPOH elements, a more profound insight in these clinical aspects are required.
